# Effects of T592 phosphomimetic mutations on tetramer stability and dNTPase activity of SAMHD1 can not explain the retroviral restriction defect

**DOI:** 10.1038/srep31353

**Published:** 2016-08-11

**Authors:** Akash Bhattacharya, Zhonghua Wang, Tommy White, Cindy Buffone, Laura A. Nguyen, Caitlin N. Shepard, Baek Kim, Borries Demeler, Felipe Diaz-Griffero, Dmitri N. Ivanov

**Affiliations:** 1 Department of Biochemistry, University of Texas Health Science Center, San Antonio, TX 78229, USA; 2 Department of Microbiology and Immunology, Albert Einstein College of Medicine, Bronx, NY 10461, USA; 3 Center for Drug Discovery, Department of Pediatrics, Emory School of Medicine, Atlanta, GA 30322, USA; 4 School of Pharmacy, Kyunghee University, Seoul, South Korea

## Abstract

SAMHD1, a dNTP triphosphohydrolase, contributes to interferon signaling and restriction of retroviral replication. SAMHD1-mediated retroviral restriction is thought to result from the depletion of cellular dNTP pools, but it remains controversial whether the dNTPase activity of SAMHD1 is sufficient for restriction. The restriction ability of SAMHD1 is regulated in cells by phosphorylation on T592. Phosphomimetic mutations of T592 are not restriction competent, but appear intact in their ability to deplete cellular dNTPs. Here we use analytical ultracentrifugation, fluorescence polarization and NMR-based enzymatic assays to investigate the impact of phosphomimetic mutations on SAMHD1 tetramerization and dNTPase activity *in vitro*. We find that phosphomimetic mutations affect kinetics of tetramer assembly and disassembly, but their effects on tetramerization equilibrium and dNTPase activity are insignificant. In contrast, the Y146S/Y154S dimerization-defective mutant displays a severe dNTPase defect *in vitro*, but is indistinguishable from WT in its ability to deplete cellular dNTP pools and to restrict HIV replication. Our data suggest that the effect of T592 phosphorylation on SAMHD1 tetramerization is not likely to explain the retroviral restriction defect, and we hypothesize that enzymatic activity of SAMHD1 is subject to additional cellular regulatory mechanisms that have not yet been recapitulated *in vitro*.

Human SAMHD1 protein is an innate immunity factor involved in the regulation of interferon signaling and retroviral restriction. Mutations in the SAMHD1 gene result in the Aicardi-Goutières syndrome (AGS), a genetic disorder caused by pathologic activation of interferon signaling^1^. In addition, SAMHD1 restricts replication of HIV-1, HIV-2 and SIV in macrophages, dendritic and resting CD4+ T cells and its restriction activity is attenuated in HIV-2 and SIV infection by the viral accessory protein Vpx, which promotes proteasomal degradation of SAMHD1^2^,^3^,^4^,^5^,^6^.

SAMHD1 is a member of the HD-domain family of hydrolases and catalyzes hydrolysis of deoxynucleotides to triphosphate and unphosphorylated nuocleosides: dNTP→dN+PPP. Following discovery of this unusual dNTPase activity in bacterial HD proteins, it was proposed that these enzymes contribute to deoxynucleotide metabolism and homeostasis^7^,^8^,^9^. Studies of SAMHD1 confirmed that the enzyme acts as a key regulator of dNTP pools during cell cycle in mammalian cells^10^. The observation that SAMHD1 activity is critical for maintaining low dNTP concentrations in monocyte-derived macrophages was particularly important. SAMHD1 blocks retroviral replication prior to completion of reverse transcription^11^,^12^ and the SAMHD1-mediated decrease in the dNTP levels in myeloid cells correlates with the inability of lentiviruses to undergo reverse transcription^13^,^14^. Thus, a model emerged, in which the restriction of retroviral replication results from the reduction of cellular dNTP concentrations below the level required for successful reverse transcription of the genome by the viral reverse transcriptase.

Recent data suggest that the retroviral restriction mechanism is likely to be more complex. Several groups discovered that SAMHD1 is regulated in cells by phosphorylation of threonine 592 (T592) located in the C-terminal segment of the HD domain. Phosphomimetic mutants of SAMHD1, T592D and T592E, display a puzzling phenotype: they appear to be intact in their ability to deplete cellular dNTP pools, but are unable to restrict HIV infection^15^,^16^,^17^,^18^. These findings raised questions about the exact role of dNTP depletion in the anti-retroviral activity of SAMHD1. In a separate set of studies, SAMHD1 was shown to possess nuclease activity and it was suggested that it is the nuclease activity that contributes to HIV restriction^19^,^20^. Reports of the nuclease activity are intriguing because two other proteins known to cause the Aicardi-Goutières syndrome, TREX1 and RNAse H, are nucleases^21^,^22^. However, the role of nuclease activity in SAMHD1 function remains controversial, because the nuclease activity can be separated from the protein *in vitro* by using more stringent purification protocols^23^. Finally, SAMHD1 is known to bind nucleic acids, and the interaction with RNA was found to inhibit the dNTPase activity of SAMHD1 *in vitro*^19^,^23^,^24^,^25^,^26^. Functional significance of the SAMHD1-nucleic acids interaction in HIV restriction remains unknown. It is, therefore, possible that the apparent uncoupling of dNTP depletion and HIV restriction observed in T592D and T592E mutants results from a detrimental effect of these mutations on a functional property of SAMHD1 that is distinct from the dNTPase activity.

Enzymatic activity of SAMHD1 is allosterically regulated. The enzyme can tetramerize in a nucleotide-dependent manner and crystallographic studies revealed that formation of a tight SAMHD1 tetramer requires nucleotide binding at two distinct allosteric sites, A1 and A2^27^,^28^,^29^,^30^,^31^,^32^. The two sites are located in close proximity to each other at an intermonomer interface within the tetramer. Site A1 is specific for guanine nucleotides and can accommodate both GTP and dGTP, whereas the A2 site is specific for deoxynucleotides, but its base specificity is not pronounced. It was suggested that the tetramer is the active form of the enzyme and that the dependence of tetramerization on dNTP binding is essential for controlling cellular dNTP concentrations. This regulatory mechanism bears some similarity to regulation of ribonucleotide reductase and thymidine kinase, two key enzymes in deoxynucleotide metabolism which are both tetrameric and allosterically controlled by nucleotide binding^33^,^34^,^35^.

Recently published studies suggested that SAMHD1 phosphorylation impacts its function through a direct effect of T592 phosphorylation on the enzymatic and allosteric properties of the protein^36^,^37^,^38^. However, the reported findings are somewhat contradictory: Tang *et al*. and Yan *et al*. observed that phosphomimetic mutations have a strong detrimental effect on protein tetramerization and cause a significant reduction in the rate of dNTP hydrolysis, whereas Arnold *et al*. found that the *k*_*cat*_ and *K*_*m*_ of the phosphorylated enzyme are comparable to that of the WT protein and proposed a more subtle mechanism, in which T592 phosphorylation interferes with the stability of the long-lived catalytically-active tetramer of SAMHD1.

In this study we investigated the effect of T592D phosphomimetic mutants on the biochemical properties of SAMHD1 using three novel experimental approaches: quantification of SAMHD1 tetramerization as a function of nucleotide concentration using analytical ultracentrifugation, characterization of allosteric properties of SAMHD1 using an NMR-based dNTPase assay and a fluorescence-polarization-based measurement of tetramer assembly and dissociation. We compare WT, phosphomimetic and tetramerization-defective mutations of SAMHD1 and conclude that their retroviral restriction phenotypes can not be explained by their ability to tetramerize or hydrolyze dNTPs *in vitro*. Further investigation of the cellular control of SAMHD1 activity is needed in order to establish mechanistic links between the biochemical, biophysical and structural properties of SAMHD1 and its function in retroviral restriction.

## Results

### Tetramerization propensity of T592D is similar to that of WT SAMHD1

**Figure 1:**
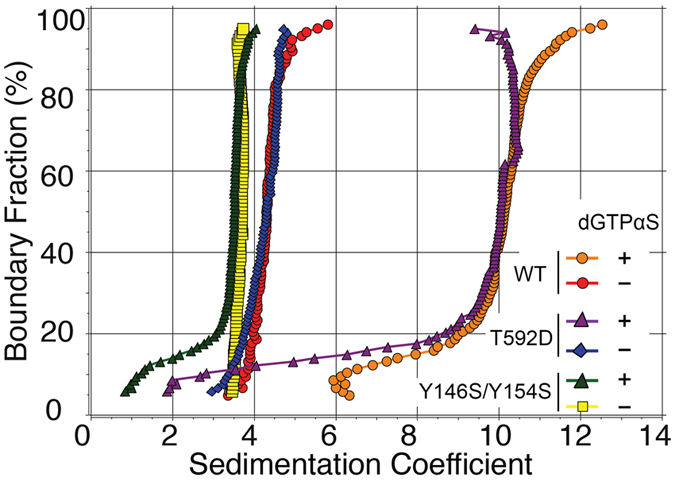
Effect of T592D and Y146S/Y154S mutations on SAMHD1_115-626_ tetramerization in the presence of dGTPαS. Model-free van Holde – Weischet analysis of the sedimentation velocity analytical ultracentrifugation experiments. The samples shown are as follows: 5.75 μM SAMHD1_115-626_ WT + 0 μM dGTPαS (red circles); 5.75 μM SAMHD1_115-626_ WT + 60 μM dGTPαS (orange circles); 5.5 μM SAMHD1_115-626_ T592D + 0 μM dGTPαS (blue diamonds); 5.5 μM SAMHD1_115-626_ T592D + 50 μM dGTPαS (purple triangles); 5.0 μM SAMHD1_115-626_ Y146S/Y154S + 0 μM dGTPαS (yellow squares); 5.0 μM SAMHD1_115-626_ Y146S/Y154S + 50 μM dGTPαS (green triangles).

AUC is the method of choice for quantitative studies of protein oligomerization. The method is well suited for studies of SAMHD1 tetramerization as the monomeric and tetrameric species are clearly distinguishable and readily quantified by van Holde-Weischet (vHW )analysis of the sedimentation velocity data^39^. For the AUC studies described here we used bacterially expressed HD domain constructs of SAMHD1 (aa 115-626), as these constructs have previously been shown to recapitulate wild-type dNTPase activity, tetramerization and HIV restriction^26^,^30^. In the absence of nucleotides the HD domain construct SAMHD1_115-626_ sediments at approximately 4 S, close to the theoretical value expected for a 60 kDa globular protein. SAMHD1 tetramerization is induced by binding of nucleotides at two allosteric sites, A1 and A2. For example, addition of dGTP induces tetramerization because this nucleotide can be accommodated at both, the A1 and A2. dGTP is also a substrate of the enzyme and its hydrolysis product, deoxyguanosine (dG), does not support SAMHD1 tetramerization, an issue that complicates AUC studies of the catalytically active protein. We and others have previously shown that dGTPαS, a dGTP analogue which is hydrolyzed at a slower rate than dGTP, can be used to investigate SAMHD1 tetramerization in sedimentation velocity experiments^39^,^40^. Upon addition of 60 μM of dGTPαS to 6 μM SAMHD1_115-626_ WT a major fraction of the protein sediments at approximately 10 S, consistent with the 240 kDa tetramer (Fig. 1). In contrast, the tetramerization defective mutant Y146S/Y154S that we previously described, remains strictly monomeric in the presence of 50 μM dGTPαS^39^. Addition of dGTPαS to the T592D mutant of the HD domain, also results in the appearance of the 10 S species, which indicates that this phosphomimetic mutant is tetramerization competent. However, there are clear deviations from the vertical in the appearance of the vHW plots for the higher molecular weight species, which is indicative of the graduate tetramer dissociation occurring during the run as dGTPαS is depleted by the dNTPase activity of SAMHD1. The data reveal that T592D can tetramerize in the presence of dGTPαS, but a more quantitative comparison of the WT and T592D tetramerization properties is not possible because of dGTPαS hydrolysis during the experiment.

**Figure 2:**
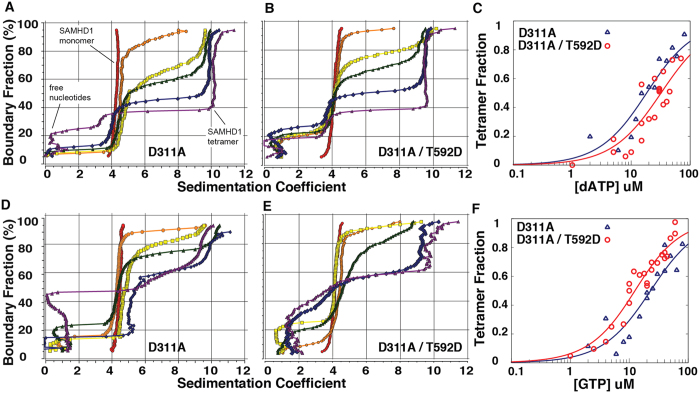
Tetramerization of SAMHD1_115-626_ D311A and D311A/T592D as a function of nucleotide concentration. Model-free van Holde – Weischet analysis of the sedimentation velocity experiments for the dATP (**A**–**C**) and GTP (**D**–**F**) titration series performed on SAMHD1_115-626_ D311A (**A**,**D**) and SAMHD1_115-626_ D311A/T592D (**B**,**E**). (**A**) dATP concentrations (μM): 0 (red); 10 (orange); 15 (yellow); 20 (green); 30 (blue); 80 (purple). (**B**) dATP concentrations (μM): 0 (red); 1 (orange); 8 (yellow); 10 (green); 24 (blue); 44 (purple). (**D**) GTP concentrations (μM): 0 (red); 6 (orange); 10 (yellow); 12 (green); 20 (blue); 50 (purple). (**E**) GTP concentrations (μM): 0 (red); 8 (orange); 10 (yellow); 15 (green); 25 (blue); 35 (purple). (**C**,**F**) Tetramer fraction plotted as a function of nucleotide concentration from multiple sedimentation velocity experiments for D311A SAMHD1_115-626_ (in blue) and D311A T592D SAMHD1_115-626_ (in red). The curves show the fit of the data to a simple EC_50_ equation y = x/(EC_50_+x).

Effect of the T592D mutation on tetramer formation can be better evaluated using catalytically dead mutants of SAMHD1^37^. We prepared HD domain constructs with a D311A mutation, which removes one of the metal coordination residues in the active site and results in the loss of dNTPase activity^20^,^41^. Tetramerization of the D311A mutant can be evaluated quantitatively by measuring the tetramer fraction as a function of the allosteric ligand concentration. GTP is the most likely physiological ligand of the A1 site and it does not bind to A2^40^,^42^, so we first investigated SAMHD1 tetramerization in the presence of 10 μM GTP and increasing amounts of dATP as the A2 ligand. The data shown in Fig. 2A shows several representative dATP concentration points from multiple titration experiments. In the presence of GTP alone SAMHD1 remains monomeric: the vHW plot reveals that the sample sediments as single species with sedimentation coefficient close to 4. As dATP concentration is increased appearance of the vHW plots become more complex. First, there is contribution to total absorbance from free nucleotides that sediment with sedimentation coefficient close to zero. In addition, as dATP concentration is increased a larger fraction of the total protein sediments as tetramer. Tetramer fraction can be quantified (see Methods) and when plotted as a function of dATP concentration the half-rise point falls close to 20 μM dATP (Fig. 2C). When dATP titration series are performed with the D311A/T592D double mutant, a very similar dependence of tetramerization on dATP concentration is observed (Fig. 2B,C).

The experiment was also performed by holding dATP concentration constant and gradually increasing GTP concentration (Fig. 2D–F). Once again no SAMHD1 tetramer was detected in the presence of dATP alone and upon addition of GTP the tetramer fraction is increased with an apparent half rise at approximately 20 μM GTP. In these titration series the differences between D311A and D311A/T592D are not significantly larger than the experimental error. Collectively these quantitative comparisons of the tetramerization propensity of SAMHD1 constructs reveal that the differences in the tetramerization ability of the phosphomimetic mutant and WT SAMHD1 are less than two fold and are insignificant compared to the dramatic tetramerization defect observed for the Y146A/Y154A mutant as previously described^39^.

Finally, we used size-exclusion chromatography as an independent experimental method to compare tetramerization of SAMHD1_115-626_ D311A and D311A/T592D constructs. Size-exclusion chromatography results are in agreement with our analytical centrifugation data and show no significant differences between D311A and D311A/T592D proteins (Fig S8).

### NMR-based dNTPase assay as a probe of SAMHD1 allosteric properties

The dNTPase activity of SAMHD1 is greatly enhanced in the presence of guanine-base nucleotides, dGTP or GTP. The mechanism of this regulation is believed to arise from the dependence of SAMHD1 dNTPase activity on protein tetramerization, which in turn depends on binding of a guanine-base nucleotide at the A1 allosteric site^27^,^28^,^40^,^41^,^42^. Measurement of dNTPase activity, therefore, offers an alternative experimental strategy for assessing allosteric and tetramerization properties of SAMHD1.

**Figure 3:**
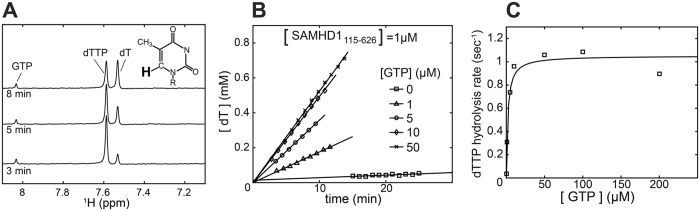
NMR-based dNTPase assay. (**A**) NMR signals of the H6 proton of the thymine base (shown in bold) display distinct chemical shift in thymidine triphosphate (dTTP) and unphosphorylated thymidine nucleoside (dT). NMR spectra are acquired at regular time intervals following addition of SAMHD1. (**B**) Reaction progress curves are obtained by plotting NMR signal intensities as a function of time. Progress curves obtained at different GPT concentrations are shown. Steady-state rates are determined by linear fitting. (**C**) The plot of the reaction rate versus GTP concentration reveals dependence of the enzymatic activity on the binding of GTP to the A1 allosteric site. The curve shows the fit of the data to a simple EC_50_ equation y = y_max_ *x/(EC_50_+x).

To facilitate measurements of dNTPase activity we have developed an NMR-based assay that allows automated, real-time, quantitative measurement of dNTP hydrolysis. The assay uses NMR intensity measurements of the nucleobase aromatic proton signals, the chemical shifts of which are sensitive to dNTP hydrolysis (Fig. 3A). The small-volume cryoprobe allows acquisition of proton NMR spectra on 30 μL samples containing 10–1000 μM concentrations of dNTPs within 1–3 minutes with signal-to-noise ratios sufficient for precise determination of the reaction rates. Detection sensitivity of the NMR-based assay is lower than the sensitivity of the recently developed fluorescence-based assays 43,44, but NMR detection is adequate for studies of the SAMHD1 Michaelis-Menten kinetics, because the high *Km* of the enzyme requires high substrate concentrations. Furthermore, the NMR-based detection does not require additional enzymes or reagents. The automated sample changer can move the samples in and out of the magnet allowing continuous monitoring of multiple samples over extended period of time. Progress curves for the reaction are obtained by plotting intensities of the NMR peaks corresponding to the substrate (dNTP) and the product (dN) of the reaction as a function of time (Fig. 3B). Experiments described in this study were performed with substrate present at 1 mM, which is significantly higher than the Michaelis constant of the enzyme [dTTP *K*_*m*_ ~ 100 μM^36^]. As the result, dTTP hydrolysis rate remains constant (the steady state regime) through most of the progress curve and can be determined with high precision by performing a linear fit of the progress curve data.

The NMR-based assay is particularly well suited for studies of SAMHD1 allosteric properties because it allows rapid measurement of reaction rates with good precision on multiple samples. In this study we investigated dependence of dTTP hydrolysis rate on GTP concentration as an independent quantitative way to evaluate tetramerization propensity of SAMHD1 mutants (Fig. 3C). GTP is preferred over dGTP in this experiment because it is not hydrolyzed by the enzyme and does not compete with the substrate for the active site. The amount of the catalytically-active tetramer is determined by coupled equilibria that include GTP binding at the A1 site and protein tetramerization. As the result, a protein tetramerization defect should manifest itself as a slower rise of the dNTP hydrolysis rate as a function of GTP concentration.

### GTP-dependence of the T592D dNTPase activity reveals no tetramerization defect

**Figure 4:**
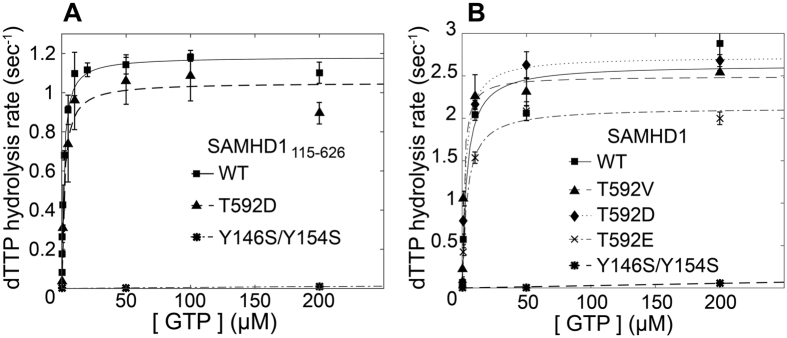
Phosphomimetic mutations have a minor effect on the dNTPase activity of SAMHD1 in contrast to the Y146S/Y154S tetramerization-defective mutant. (**A**) dNTPase activity as a function of GTP concentration measured for bacterially-expressed SAMHD1_115-626_ HD domain constructs. (**B**) dNTPase activity as a function of GTP concentration measured for full-length SAMHD1 constructs expressed in mammalian cells.

The rate of dTTP hydrolysis by the WT SAMHD1 HD domain construct displays strong dependence on GTP concentration rising from < 0.1 sec^−1^ with no GTP added to > 1.0 sec^−1^ at GTP concentrations above 20 μM with the apparent half-rise point at 1.5 μM GTP (Fig. 4A). HD domain containing T592D mutation displays very similar GTP dependence with the apparent half-rise at 2.0 μM GTP. In contrast, the tetramerization defective mutant displays a strong dNTPase defect with apparent dTTP hydrolysis rate below 0.05 sec^−1^ even at 200 μM GTP. These results are in agreement with our AUC studies and indicate that the effect of the phosphomimetic mutation T592D is minor if at all present, in contrast to Y146A/Y154A, which results in a major tetramerization and dNTPase defect *in vitro*.

It is possible that the impact of the phosphomimetic mutation on the tetramerization properties and dNTPase activity of SAMHD1 may not manifest itself in the bacterially expressed protein and may involve additional post-translational modifications, chaperonin activities or other factors not present in bacteria. To evaluate this possibility we expressed full-length, FLAG-tagged SAMHD1 in human 293T cells, purified it using anti-FLAG agarose beads and investigated its dNTPase activity using the NMR-based assay (Fig. 4B). Protein concentrations of SAMHD1 constructs were quantified as explained in *Methods*. We observed that SAMHD1 constructs purified from mammalian cells consistently displayed 2–2.5 fold higher activities than the bacterially expressed HD-domain constructs. This observation suggests that SAMHD1 biochemistry may not be fully recapitulated in a bacterially expressed HD domain. However, relative dNTPase activities of the mutant constructs display the same pattern as their bacterially-expressed HD domain counterparts. WT and T592D curves were very similar, T592E displayed ~25% lower *V*_*max*_ than WT and T592D, whereas the dimerization defective Y146A/Y154A mutant was dramatically less active. Collectively, our dNTPase activity studies indicate that phosphomimetic mutations do not have a significant effect on the allosteric enhancement of SAMHD1 dNTPase activity.

### T592D phosphomimetic mutant displays faster kinetics of SAMHD1 tetramer dissociation

A recent study suggested that phosphomimetic mutations may affect the ability of SAMHD1 to form “long-lived” tetramers, which may be required for depletion of dNTPs to sufficiently low levels. To evaluate whether phosphomimetic mutations affect kinetics of tetramer formation and dissociation we developed a fluorescence polarization based method to monitor tetramerization kinetics. The method uses GTP with a fluorescent tag attached to its gamma phosphate (see *Methods*) which is herein referred to as GTP-F. Free GTP-F tumbles rapidly in solution, which results in low polarization values observed in a fluorescence polarization measurement. When GTP-F binds at the A1 site of SAMHD1 tetramer a significant increase in the observed fluorescence polarization is expected.

**Figure 5:**
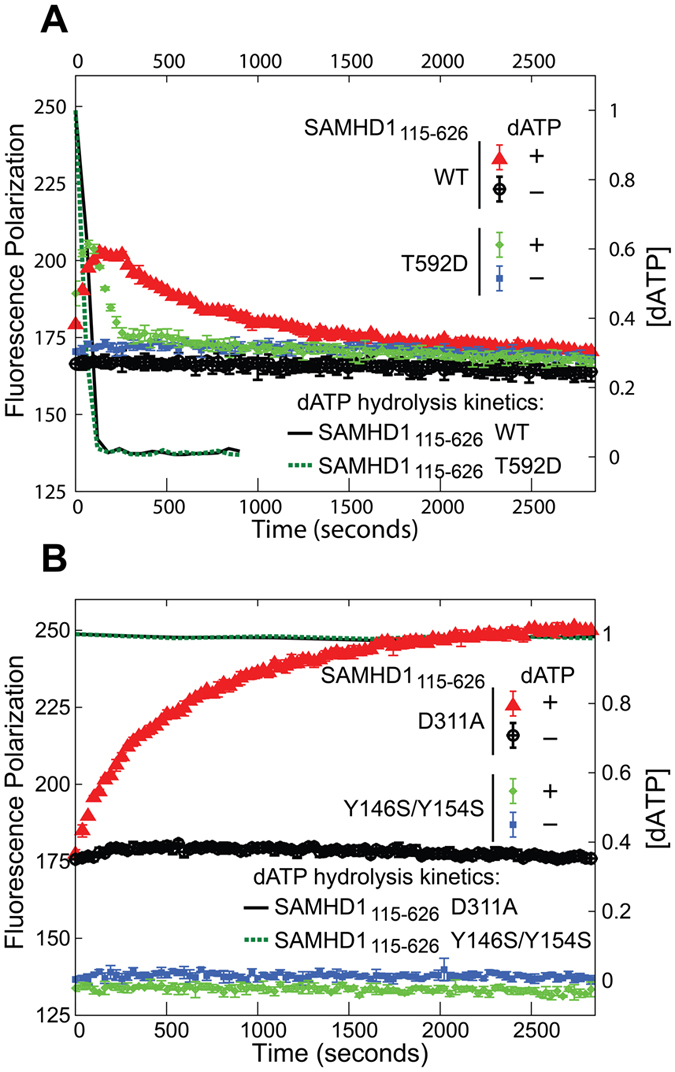
Kinetics of SAMHD1_115-626_ tetramer association/dissociation. Fluorescence polarization (left Y axis) measured as a function of time following addition of 0.5 mM dATP to SAMHD1_115-626_ constructs. (**A**) Catalytically competent SAMHD1_115-626_ WT and T592D. (**B**) Catalytically inactive SAMHD1_115-626_ D311A and Y146S/Y154S. Kinetics of dATP depletion (right Y axis) was measured for these samples using NMR-based dNTPase assay and plotted as solid black and dashed green lines.

Experiments were performed in 384-well microplates with samples containing equimolar amounts of SAMHD1 and unlabeled GTP spiked with a small amount of GTP-F. At time t = 0 the samples were injected either with dATP stock solution or with buffer to yield the following final concentrations of the reagents: 3 μM SAMHD1, 3 μM GTP, 20 nM GTP-F and either 500 μM or 0 μM dATP. Fluorescence polarization measurements were then taken every 31 seconds (Fig. 5).

When no dATP is added, fluorescence polarization readings remain roughly constant in all samples studied throughout the 1-hour experiment. When dATP is added to the WT protein, an increase in fluorescence polarization is observed (Fig. 5A). Polarization value increases from 170 to 200 polarization units in approximately 3 minutes and then starts to decay back to its baseline value with the half-life of approximately 10 minutes. In contrast to WT protein, the tetramerization-defective Y146S/Y154S mutant shows no change in fluorescence polarization after addition of dATP confirming that the increase in fluorescence polarization observed for WT does indeed result from protein oligomerization (Fig. 5B). When the experiment is performed with the catalytically incompetent D311A mutant, there is a rise in fluorescence polarization, but there is no subsequent decay, which confirms that the polarization decay is due to tetramer dissociation occurring after dATP depletion by the WT enzyme (Fig. 5B). We also measured dATP hydrolysis in these samples using NMR. In WT and T592D samples dATP was depleted below detection sensitivity within 120 seconds, whereas no dATP hydrolysis was observed in the D311A and Y146S/Y154S controls (Fig. 5A,B, right y axis).

The phosphomimetic mutant T592D displays an increase and subsequent decay of fluorescence polarization that are similar in magnitude to that of the WT protein. However, the kinetics of tetramer formation and subsequent dissociation are different in T592D and WT proteins. Tetramer dissociation rate appears 3–5 fold faster for the T592D mutant than for WT. Furthermore, it appears that the rate of tetramer formation is also faster for T592D, although the time resolution of the experiment makes this measurement less precise. The fact that both the association and dissociation rates are increased in the T592D mutant may explain why we observe no significant difference between T592D and WT in our AUC and dNTPase experiments which measure equilibrium constants rather than rates.

### The ability of SAMHD1 mutants to lower cellular dNTP concentrations and to restrict HIV infection does not correlate with their dNTPase and tetramerization properties *in vitro*

**Figure 6:**
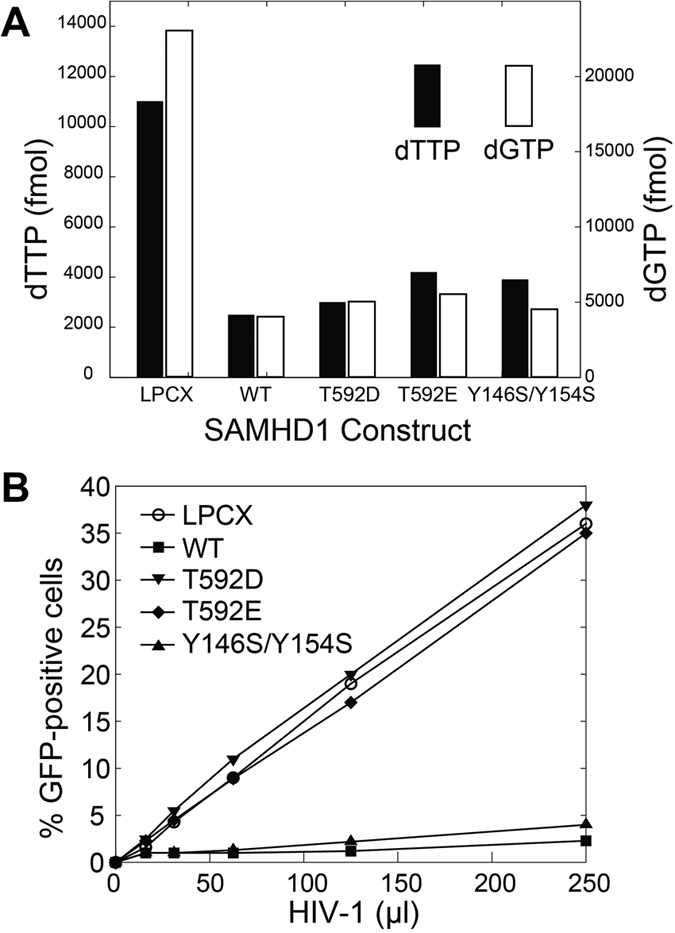
Ability of phosphomimetic and dimerization-defective SAMHD1 variants to block HIV-1 infection and decrease the cellular levels of dNTPs does not correlate with their ability to tetramerize and hydrolyze dNTPs *in vitro*. (**A**) Total cellular levels of dGTP and dTTP were measured in PMA-treated U937 cells stably expressing SAMHD1 variants by using a primer extension assay, as described in *Methods*. (**B**) PMA-treated U937 cells stably expressing the indicated SAMHD1 variants were challenged with increasing amounts of HIV-1-GFP. Infection was determined by measuring the percentage of GFP-positive cells using flow cytometry.

Finally, we compared the ability of the phosphomimetic (T592D and T592E) and tetramerization-defective (Y146S/Y154S) mutants to deplete cellular dNTP pools and to restrict HIV infection. For this purpose, we stably expressed the indicated full-length SAMHD1 variants in human monocytic U937 cells. Cellular levels of dNTPs were measured in PMA-treated U937 cells expressing the different SAMHD1 variants, as previously described^18^,^26^ (Fig. 6A). In agreement with previous results, all tested variants retained the ability to decrease the cellular levels of dNTPs^18^,^39^. We also tested the ability of the different SAMHD1 variants to block HIV-1 infection (Fig. 6B). To this end, PMA-treated U937 cells stably expressing the different SAMHD1 variants were challenged with increasing amounts of HIV-1 virus expressing GFP as a reporter of infection. As shown in Fig. 6B, SAMHD1 phosphomimetic mutants T592D and T592E lost the ability to block HIV-1 infection, which is in agreement with previous observations^18^. On the contrary, the SAMHD1 oligomerization deficient mutant Y146S/Y154S restricted HIV-1 infection, as previously shown^39^.

## Discussion

SAMHD1-mediated inhibition of retroviral replication is an important component of antiretroviral defenses as illustrated by targeted depletion of SAMHD1 by simian immunodeficiency viruses and HIV-2; however, the mechanism of SAMHD1-mediated retroviral restriction is not fully understood. Early studies suggested that the dNTPase activity of the enzyme depletes cellular dNTP concentrations and thus inhibits reverse transcription of the viral genome^5^,^6^,^12^,^14^. The discovery that T592 phosphorylation regulates retroviral restriction raised questions about the exact role of dNTPase activity in restriction because phosphomimetic mutants of SAMHD1 can deplete the cellular levels of dNTPs, but do not inhibit viral replication^15^,^16^,^17^,^18^.

dNTPase activity of SAMHD1 depends on protein tetramerization mediated by binding of nucleotides at allosteric sites. It was suggested by recent studies that the restriction defect of phosphomimetic mutations can be explained by a direct effect of these mutations on protein tetramerization^36^,^37^,^38^. Our results do not support this simple model.

The analytical ultracentrifugation methodology we employ allows for quantitative comparison of tetramerization properties of SAMHD1 HD domain constructs. We do not observe any significant effects of the T592D mutation on the ability of SAMHD1 to tetramerize in the presence of dATP and GTP when we measure the amount of SAMHD1 tetramer as a function of nucleotide concentration. The ultracentrifugation studies provide another novel insight: the experiments reveal that in the presence of only one allosteric ligand, the protein remains predominantly monomeric even at high protein and ligand concentrations. With the addition of the second allosteric ligand we observe that an increasing fraction of the protein sediments as tetramer, with no observable accumulation of the dimeric species. Our data indicate that SAMHD1 oligomerization is a process, in which allosteric ligand binding, dimerization and tetramerization are concerted events that convert the inactive monomeric SAMHD1 into the catalytically-active tetramer. In a concerted process, defects that affect any of the protein-protein interfaces involved in SAMHD1 tetramerization should affect the dependence of tetramer fraction on nucleotide concentration. Since we do not observe any such effect with the T592D mutant, it suggests that the structural differences observed in the crystal structures of phosphomimetic mutants^37^ do not contribute significantly to ability of SAMHD1 to tetramerize in the presence of nucleotides.

Results of the NMR-based dNTPase studies further support the analytical ultracentrifugation data. We find that the dependence of the catalytic rate on GTP concentration is not significantly affected by phosphomimetic mutations neither in the bacterially-expressed HD domain constructs, nor in the full-length constructs purified from mammalian cells. Once again, a deleterious effect of phosphomimetic mutants on the stability of SAMHD1 tetramer should manifest itself as a slower rise of the catalytic rate vs GTP concentration curve, which we do not observe in our experiments.

These results do not support the model of a direct detrimental effect of phosphomimetic mutations on tetramer stability and dNTPase activity of SAMHD1 proposed by Tang *et al*. and Yan *et al*.^37^,^38^. Our dNTPase data are in agreement with the results of Arnold at al., who also observed no significant change in the *K*_*m*_ and *k*_*cat*_ of the phosphorylated HD domain of SAMHD1. Arnold at al. suggested that phosphorylation has a more subtle effect on the enzymatic activity of SAMHD1. They argue that T592 phosphorylation impairs the ability of SAMHD1 to form a “long-lived”, enzymatically active tetramer and, as a result, the phosphorylated enzyme is not able to deplete dNTPs to sufficiently low concentrations needed to inhibit viral replication. In agreement with Arnold at al., our fluorescence polarization studies reveal that in the T592D construct tetramer dissociation that follows dNTP depletion is indeed 3–5 fold faster than in the WT construct.

Whereas the proposed role of the long-lived, enzymatically-active SAMHD1 tetramer is intriguing^36^,^40^, many questions remain about the nature of this long-lived active state of the protein and its role in the restriction mechanism. Results described here and in other published studies make it unlikely that differences in the tetramer dissociation kinetics alone can account for the restriction defect of the phosphomimetic mutants and explain the mechanism linking protein phosphorylation to restriction.

We show here that the effect of the T592D mutation on tetramer lifetime is a purely kinetic effect that does not alter the tetramerization equilibrium. It is difficult to envision how a mutation that affects tetramer assembly/disassembly kinetics but not the equilibrium can result in the inability of the protein to restrict HIV replication. Kinetic differences can, in principle, produce distinct transient effects in dNTP concentrations, but they can not explain how the wild-type protein gains a sustained ability to restrict viral replication upon dephosphorylation in long-lived non-cycling cells whereas the phosphomimetic mutant does not.

Furthermore, in agreement with our *in-vitro* observations, the phosphomimetic mutations do not have any effect on the ability of the enzyme to deplete total cellular dNTP pools^18^, as measured by a primer extension assay^13^,^14^,^45^. The models proposed by Tang *et al*. or Arnold at al. do not address this surprising observation. One can hypothesize that the primer extension assay^45^ does not fully capture some spatial, temporal or yet another aspect of SAMHD1-mediated dNTP depletion in cells. However, it remains unknown what aspect of dNTP depletion in cells may potentially be phosphorylation sensitive and critical for the ability of the enzyme to restrict retroviral replication.

Another puzzling question is how an enzyme with *k*_*cat*_ < 10 sec^−1^ and *K*_*m*_ of approximately 100 μM^36^,^40^ can deplete cellular dNTP concentrations below 100 nM on a physiologically-relevant time scale. *K*_*m*_ and *k*_*cat*_ values measured *in vitro* suggest that we may be missing an important part of how SAMHD1 activity is regulated in cells.

Finally, the tetramerization-defective Y146S/Y154S mutant of SAMHD1 provides another striking illustration that our understanding of the mechanism linking SAMHD1 tetramerization and dNTPase activity *in vitro* to retroviral restriction in cells is at best incomplete. In agreement with our previously published study, Y146S/Y154S displays a major tetramerization and dNTPase defect *in vitro*^39^. Remarkably, the Y146S/Y154S mutant is virtually indistinguishable from WT protein in its ability to deplete cellular dNTP pools and restrict HIV infection. The Y146S/Y154S data suggest that the dNTPase activity in cells may either not require protein tetramerization, or that the tetramerization defect observed *in vitro* could be compensated in cells by an unknown regulatory mechanism.

In summary, our studies of the phosphomimetic and tetramerization-defective mutants of SAMHD1 reveal poor correlation between tetramerization propensity and dNTPase activity observed *in vitro* and the ability of the proteins to deplete cellular dNTPs and to restrict retroviral restriction. We observe that effects of phosphomimetic mutations on the tetramerization equilibrium and dNTPase activity of SAMHD1 are minor and can not explain the strong restriction defect displayed by these mutants in cells. The impact of the T592D mutation on tetramer association/dissociation rates is intriguing, but the exact role of association/dissociation rates in the retroviral restriction mechanism requires further study. The phenotype of the Y146S/Y154S mutant raises additional questions about the role of SAMHD1 tetramerization and dNTPase activity in the retroviral restriction by SAMHD1. Collectively, the phosphomimetic mutants and the Y146S/Y154S mutant of SAMHD1 illustrate that our knowledge about the structure and biochemistry of SAMHD1 is not yet sufficient to satisfactorily explain the retroviral restriction mechanism.

Despite the issues discussed above, the key role of dNTPase activity in SAMHD1-mediated retroviral restriction is well supported by an extensive body of data. Unlike the controversial nuclease activity, the dNTPase activity has been structurally and biochemically characterized in several HD domain proteins, and mutations of key active site residues invariably result in the loss of retroviral restriction by SAMHD1^17^,^19^,^20^,^23^,^28^,^29^,^31^,^46^. One likely explanation for the puzzling phenotypes observed for phosphomimetic mutations and Y146S/Y154S is that nucleotide-dependent protein tetramerization is not the only mechanism controlling SAMHD1-mediated dNTP hydrolysis in cells. Activity of ribonucleotide reductase, the enzyme that generates most dNTPs in cells, is subject to elaborate, multi-layered regulation, of which protein multimerization is just one aspect^33^. It is possible that regulation of SAMHD1 activity in cells is similarly complex and has not been fully recapitulated *in vitro*. SAMHD1 activity in cells may be regulated by additional post-translational modifications, by interactions with other cellular or viral proteins and by nucleic acid binding. *In-vitro* biochemical properties should be correlated with cellular phenotypes for a larger selection of SAMHD1 mutants in order to establish mechanistic links between biochemical, biophysical and structural properties of SAMHD1 and its function in retroviral restriction.

## Methods

### Protein expression and purification

Plasmids for bacterial expression of SAMHD1 HD domain constructs were prepared by cloning codon-optimized DNA sequence encoding human SAMHD1 (residues 115-626) into pET30a vector with an N-terminal Strep-tag^39^. All mutants were generated using QuikChange Lightning mutagenesis kit (Agilent). Plasmids were used to transform BL21(DE3) cells. Cells were grown at 37˚C in LB media until A_260_ ~ 0.6, transferred to 18˚C and protein expression induced with 1 mM IPTG. Cells were harvested 12–15 hours post induction. The protein was purified using Strep-Tactin affinity chromatography (IBA), and further purified using size exclusion chromatography on Hiload 16/60 Superdex 200 column (GE Life Sciences) in the following buffer: 20 mM Tris pH7.4, 150 mM NaCl, 1 mM TCEP. Protein concentrations were determined by UV absorbance at 280 nm using the theoretically calculated extinction coefficient ε = 68,760 M^−1^cm^−1^. Purified proteins were analyzed by SDS-PAGE (Fig. S1) and the identity of the mutants was confirmed by intact mass ESI-TOF (Fig. S2).

Full-length SAMHD1 protein constructs were expressed in 293T cells. Approximately 1.0 × 10^7^ human 293T cells were transfected with plasmids encoding wild type or variants SAMHD1 proteins tagged with FLAG. After 24 hours, cells were lysed in 0.5 ml of whole-cell extract (WCE) buffer (50 mM Tris pH 8.0, 280 mM NaCl, 0.5% IGEPAL, 10% glycerol, 5 mM MgCl2, 50 μg/ml ethidium bromide, 50 U/ml benzonase (Roche)). Cell debris were removed by centrifugation at 14,000 rpm for 1 h at 4 °C. Post-spin lysates were then pre-cleared using protein A-agarose (Sigma) for 1 h at 4 °C; a small aliquot of each of these lysates was stored as Input. Pre-cleared lysates containing the tagged proteins were incubated with anti-FLAG-agarose beads (Sigma) for 2 h at 4 °C. Anti-FLAG-agarose beads were washed three times in WCE buffer, and immune complexes were eluted using 200 μg/ml FLAG tripeptide in WCE buffer. The eluted samples were analyzed by SDS-PAGE and Western blotting using anti-FLAG antibodies. Protein concentrations of immunopurified full-length constructs were quantified by optical densitometry of the coomassie-stained SDS-PAGE gels using the bacterially-expressed HD domain construct as a standard (Fig. S3).

### Analytical ultracentrifugation

SAMHD1 samples were prepared for AUC by incubating 5 uM of protein for 20 minutes in a buffer of 50 mM TRIS, 50 mM NaCl, 5 mm MgCl2 and 2 mm DTT reducing agent at pH 8 with 10 μM of GTP and 0 to 80 μM of dATP in a titration series. The UV-absorbance were checked to ensure that A280 did not exceed 1.0 for the final titration point (corresponding to ~80 uM dATP). 450 μM samples were sedimented at 50 krpm and 20 °C in a Beckman Optima XLA-1 centrifuge equipped with a 8-hole An50-Ti rotor.

The data was analyzed by 2D-spectrum analysis using the Ultrascan III software^47^,^48^. Ultrascan III performs a two-dimensional grid search over user-defined ranges of sedimentation rate and anisotropy to solve a system of Lamm equations which then yields simulated absorbances that are fitted against measured absorbances. These fits and the corresponding residuals are shown in the Supplementary Figs S4–S7. Time and radially invariant noise was subtracted, and the data was subjected to model-independent van Holde – Weischet (vHW) analysis. Fractions of monomeric and tetrameric species were determined directly from the the vHW plots by calculating what fraction of total absorbance had sedimentation coefficients in the 2–7 range (monomer) and above 7 (tetramer).

### NMR-based dNTPase assay

All 1D NMR spectra were collected on Bruker 500 MHz instrument equipped with 1.7 mm micro cryo-probe and a SampleJet automated sample changer. NMR samples were prepared as follows: 1 mM dTTP substrate, 1 μM SAMHD1, 10% D2O in NMR buffer (50 mM Tris, pH7.4, 150 mM NaCl, 5 mM MgCl_2_ and 5 mM DTT). ^1^H 1D NMR spectra were recorded at regular time intervals and were processed using NMRPipe {Delaglio, 1995 #86}. Relative NMR peak intensities of the H6 proton signal of deoxythymidine triphosphate versus deoxythymidine nucleoside were used to determine substrate and product concentrations as a function of time (Fig. 3). NMR peak amplitudes were used as a measure of peak intensities. Peak maxima were identified using a peak maximum search functionality in NMRPipe. The sum of substrate and product intensities was normalized to unity to remove noise arising from variations in detection sensitivity. Hydrolysis rates were determined by linear fitting of the reaction progress curves. Progress curve analysis, curve fitting and data plotting were performed using MATLAB software (Mathworks).

### Fluorescence polarization studies of tetramer assembly/disassembly kinetics

Kinetics of SAMHD1 tetramer assembly was investigated using fluorescence polarization. The reporter molecule was g-(6-Aminohexyl)-GTP-ATTO-495 (Jena Biosciences Cat # NU-834-495), referred to as GTP-F in this article. We tested the binding of GTP-F to various constructs of SAMHD1 by measuring samples in triplicate in a 384-well microplate. Fluorescence polarization measurements were performed on a BMG Labtech Pherastar FS multimode plate reader. Measurements were performed after injecting buffer or dATP stock solutions into protein samples as described in the Results section. All samples were prepared in the buffer containing 50 mM Tris pH 8.0, 50 mM NaCl, 5 mm MgCl2 and 2 mm DTT. Fluorescence Polarization readings were acquired at 31 second intervals for 3700 seconds.

### Generation of U937 cells stably expressing SAMHD1 variants

Human U937 (ATCC#CRL-1593) cells were grown in RPMI suplemented with 10% (v/v) fetal bovine serum and 1% (v/v) penicilin/streptomycin. LPCX-SAMHD1-FLAG plasmids expressing the codon optimized SAMHD1 fused to either FLAG epitope were previously described. The plasmids expressing human SAMHD1 polymorphisms were created using specific primers and pLPCX-SAMHD1-FLAG as template.

Retroviral vectors encoding wild type or mutant SAMHD1 proteins fused to FLAG were created using the LPCX vector (Clontech). Recombinant viruses were produced in 293T cells by co-transfecting the LPCX plasmids with the pVPack-GP and pVPack-VSV-G packaging plasmids (Clontech). The pVPack-VSV-G plasmid encodes the vesicular stomatitis virus G envelope glycoprotein, which allows efficient entry into a wide range of vertebrate cells^49^. Transduced human monocytic U937 cells were selected in 0.4 μg /ml puromycin (Sigma).

### Infection with HIV-1 expressing the green fluorescent protein (GFP)

Recombinant retroviruses expressing GFP, pseudotyped with the VSV-G glycoprotein, were prepared as described^50^. For infections, 6 × 10^4^ cells were seeded in 24-well plates and treated with 10 ng/ml phorbol-12-myristate-3-acetate (PMA) for 16 hours. PMA stock solution was prepared in DMSO at 250 μg/ml. Subsequently, cells were incubated with the indicated retrovirus for 48 hours at 37 °C. The percentage of GFP-positive cells was determined by flow cytometry (Becton Dickinson). Viral stocks were titrated by serial dilution on dog Cf2Th cells.

### Cellular dNTPs quantification by a primer extension assay

2–3 × 10^6^ cells were collected for each cell type. Cells were washed twice with 1× PBS, pelleted and resuspended in ice cold 65% methanol in Millipore grade water. Cells were vortexed for 2 minutes and incubated at 95 °C for 3 minutes. Cells were centrifuged at 14000 rpm for 3 minutes and the supernatant was transferred to a new tube for the complete drying of the methanol by using a speed vac. The dried samples were resuspended in Millipore grade water. An 18-nucleotide primer labeled at the 5′ end with a-^32^P (5′-GTCCCTGTTCGGGCGCCA-3′) was annealed at a 1:2 ratio respectively to four different 19-nucleotide templates (5′-NTGGCGCCCGAACAGGGAC-3′), where ‘N’ represents nucleotide variation at the 5′ end. Reaction condition contains 200 fmoles of template primer, 2 μl of 0.5 mM dNTP mix for positive control or dNTP cell extract, 4 μl of excess HIV-1 RT, 25 mM Tris-HCl, pH 8.0, 2 mM dithiothreitol, 100 mM KCl, 5 mM MgCl_2_, and 10 μM oligo(dT) to a final volume of 20 uL. The reaction was incubated at 37 °C for 5 minutes before being quenched with 10 uL of 40 mM EDTA and 99% (vol/vol) formamide at 95 °C for 5 minutes. The extended primer products were resolved on a 14% urea-PAGE gel and analyze using a phosphoimager. The extended products were quantified using QuantityOne software to quantify percent volume of saturation. The quantified dNTP content of each sample was accounted for based on its dilution factor, so that each sample volume was adjusted to obtain a signal within the linear range of the assay^13^,^14^,^51^.

## Additional Information

**How to cite this article**: Bhattacharya, A. *et al*. Effects of T592 phosphomimetic mutations on tetramer stability and dNTPase activity of SAMHD1 can not explain the retroviral restriction defect. *Sci. Rep.* **6**, 31353; doi: 10.1038/srep31353 (2016).

## Supplementary Material

Supplementary Figures
