# Supplementary material for: Effects of T592 phosphomimetic mutations on tetramer stability and dNTPase activity of SAMHD1 can not explain the retroviral restriction defect
Source: Sci Rep. 2016 Aug 11;6:31353. doi: 10.1038/srep31353 (PMC4980677; doi:10.1038/srep31353)
Supplement: Supplementary Figures [file srep31353-s1.pdf]

Effects of T592 phosphomimetic mutations on tetramer stability and dNTPase activity of SAMHD1 can not explain the retroviral restriction defect

AUTHORS AND AFFILIATIONS

Akash Bhattacharya[1#], Zhonghua Wang[1#], Tommy White[2], Cindy Buffone[2], Laura A Nguyen[3], Caitlin N. Shepard[3], Baek Kim[3,4], Borries Demeler[1], Felipe Diaz-Griffero[2]*, Dmitri Ivanov[1]*

[1] Department of Biochemistry, University of Texas Health Science Center, San Antonio, TX 78229

[2] Department of Microbiology and Immunology, Albert Einstein College of Medicine, Bronx, NY 10461

[3] Center for Drug Discovery, Department of Pediatrics, Emory School of Medicine, Atlanta, GA 30322

[4] School of Pharmacy, Kyunghee University, Seoul, South Korea

[#]authors contributed equally
*correspondence: ivanov@uthscsa.edu; felipe.diaz-griffero@einstein.yu.edu

SUPPLEMENTARY DATA

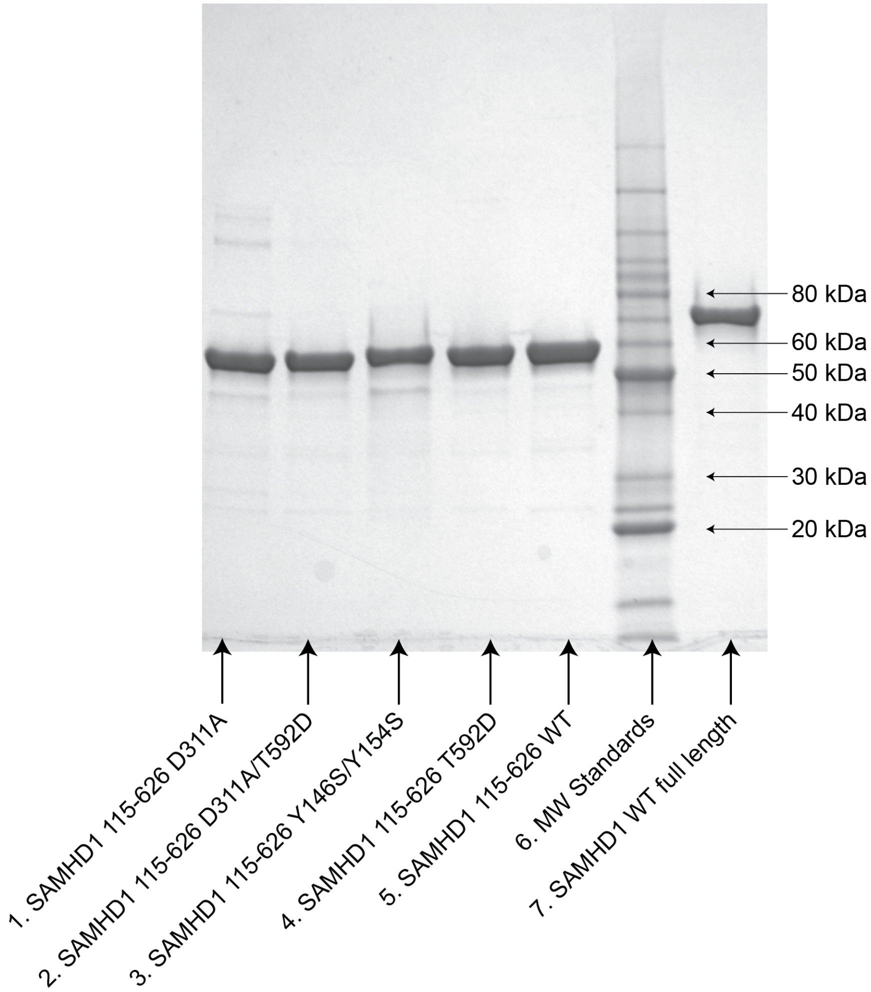

Figure S1. SDS-PAGE analysis of the bacterially expressed SAMHD1 proteins.

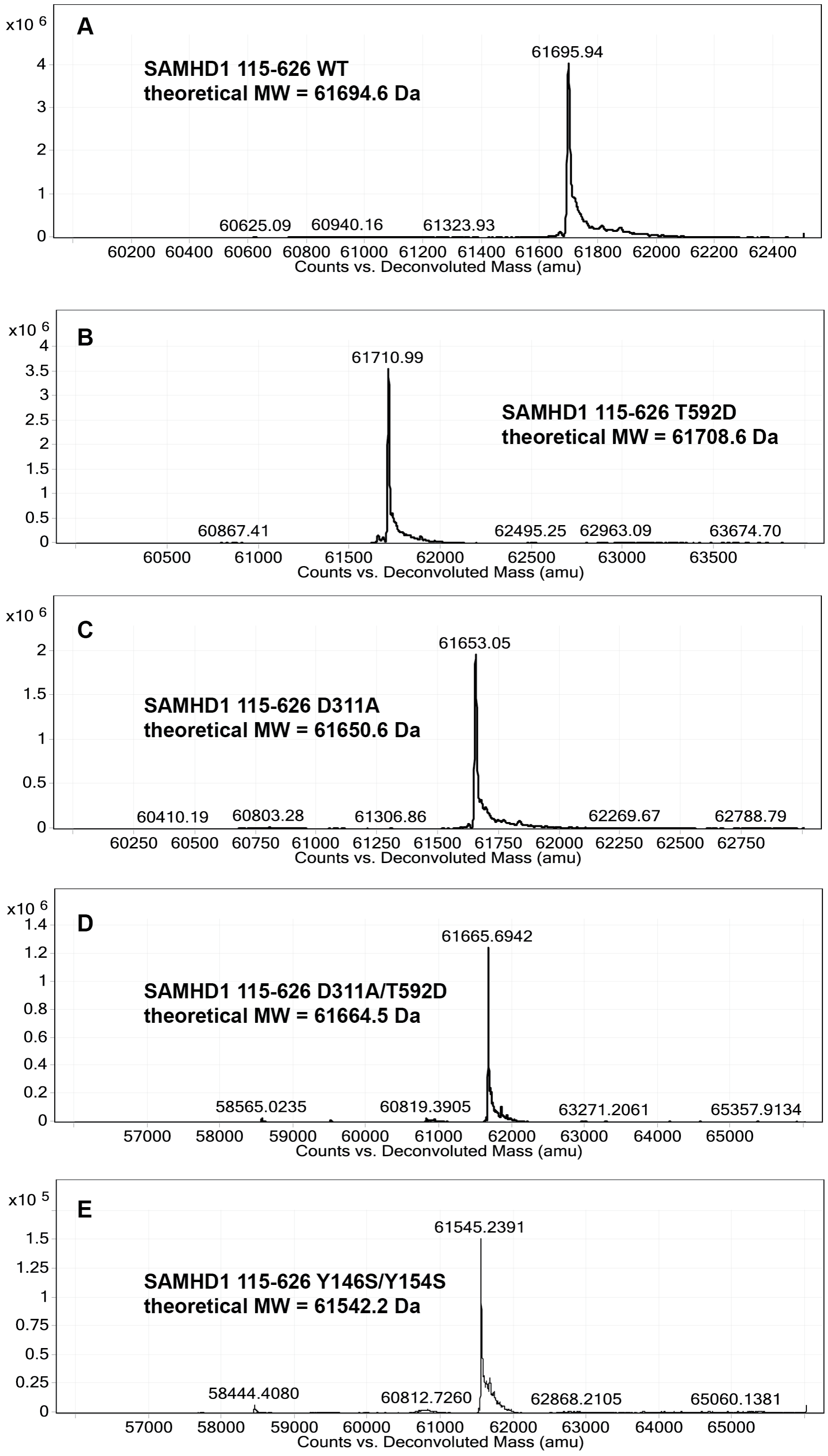

Figure S2. Intact mass ESI-TOF analysis of the bacterially expressed SAMHD1 constructs. Deconvoluted ESI-TOF mass spectra of SAMHD1 samples acquired using Agilent 6224 mass spectrometer.

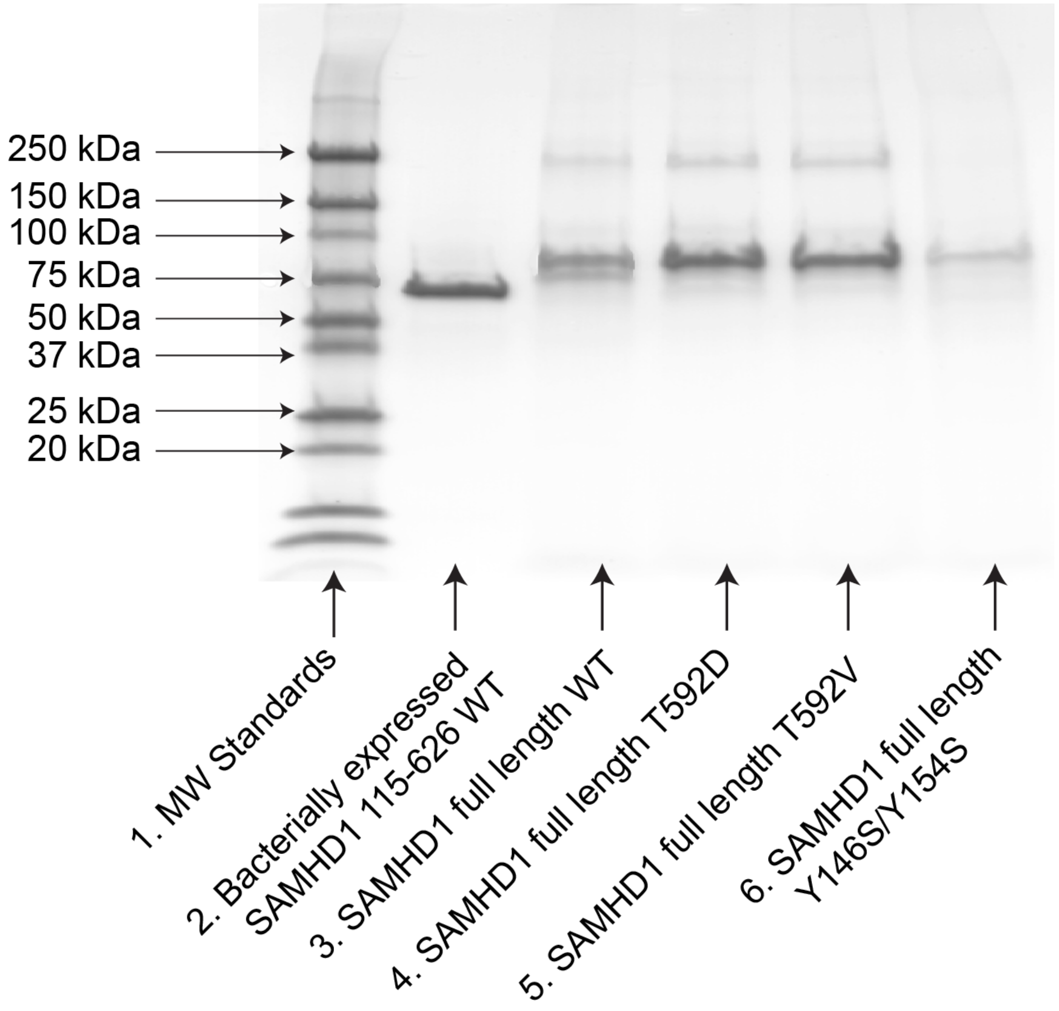

Figure S3. SDS-PAGE analysis of the full-length SAMHD1 constructs expressed in mammalian cells. Protein concentrations of immunopurified full-length constructs (lanes 3-6) were quantified by optical densitometry of the coomassie-stained SDS-PAGE gels using the bacterially-expressed HD domain construct (lane 2) as a standard.

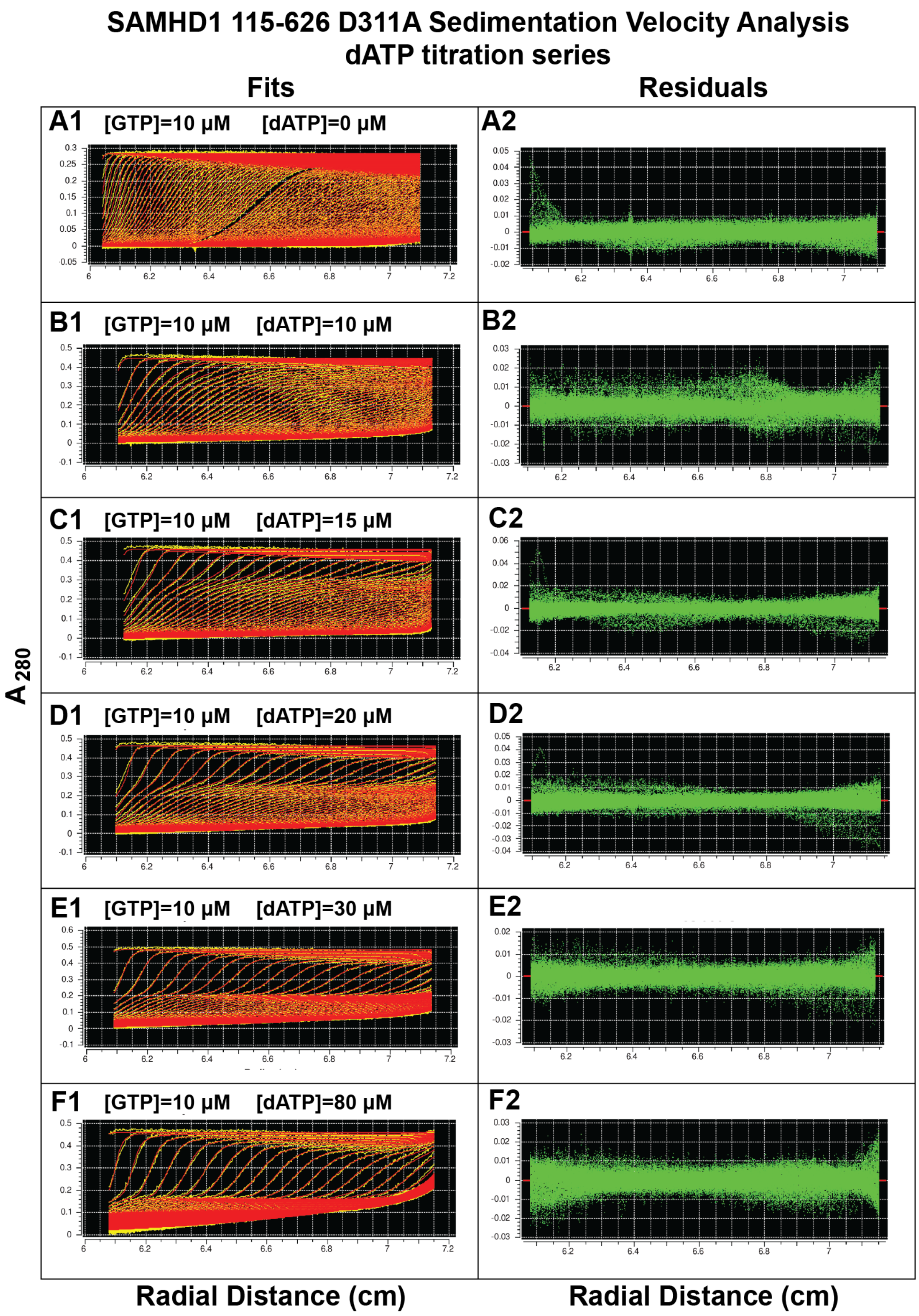

Figure S4. Sedimentation velocity analysis of SAMHD1 115-626 D311A. dATP titration series. Left column shows fits of the 2D spectrum analysis to the experimental data. Right column shows residuals for the fits shown in the left column.

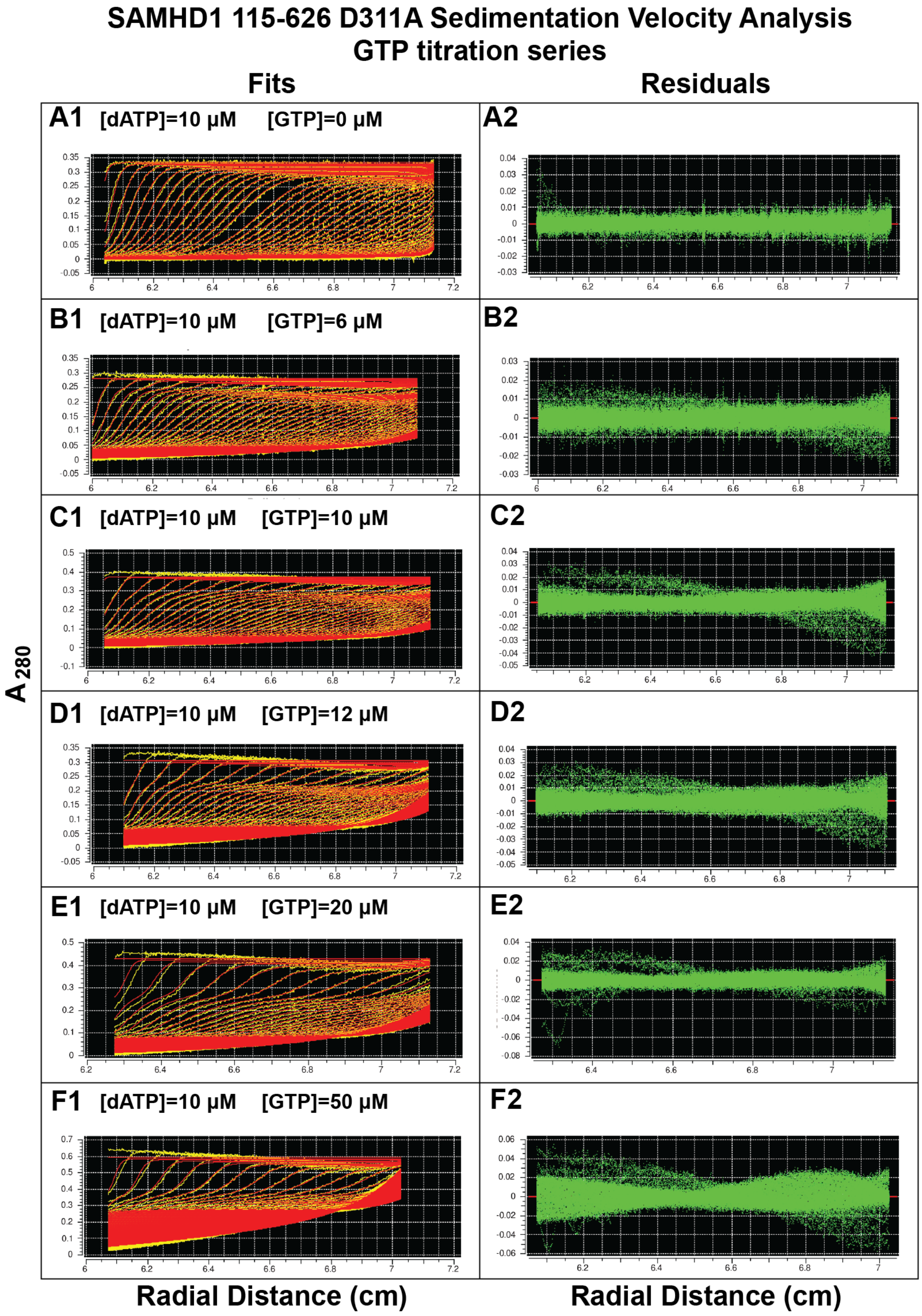

Figure S5. Sedimentation velocity analysis of SAMHD1 115-626 D311A. GTP titration series. Left column shows fits of the 2D spectrum analysis to the experimental data. Right column shows residuals for the fits shown in the left column.

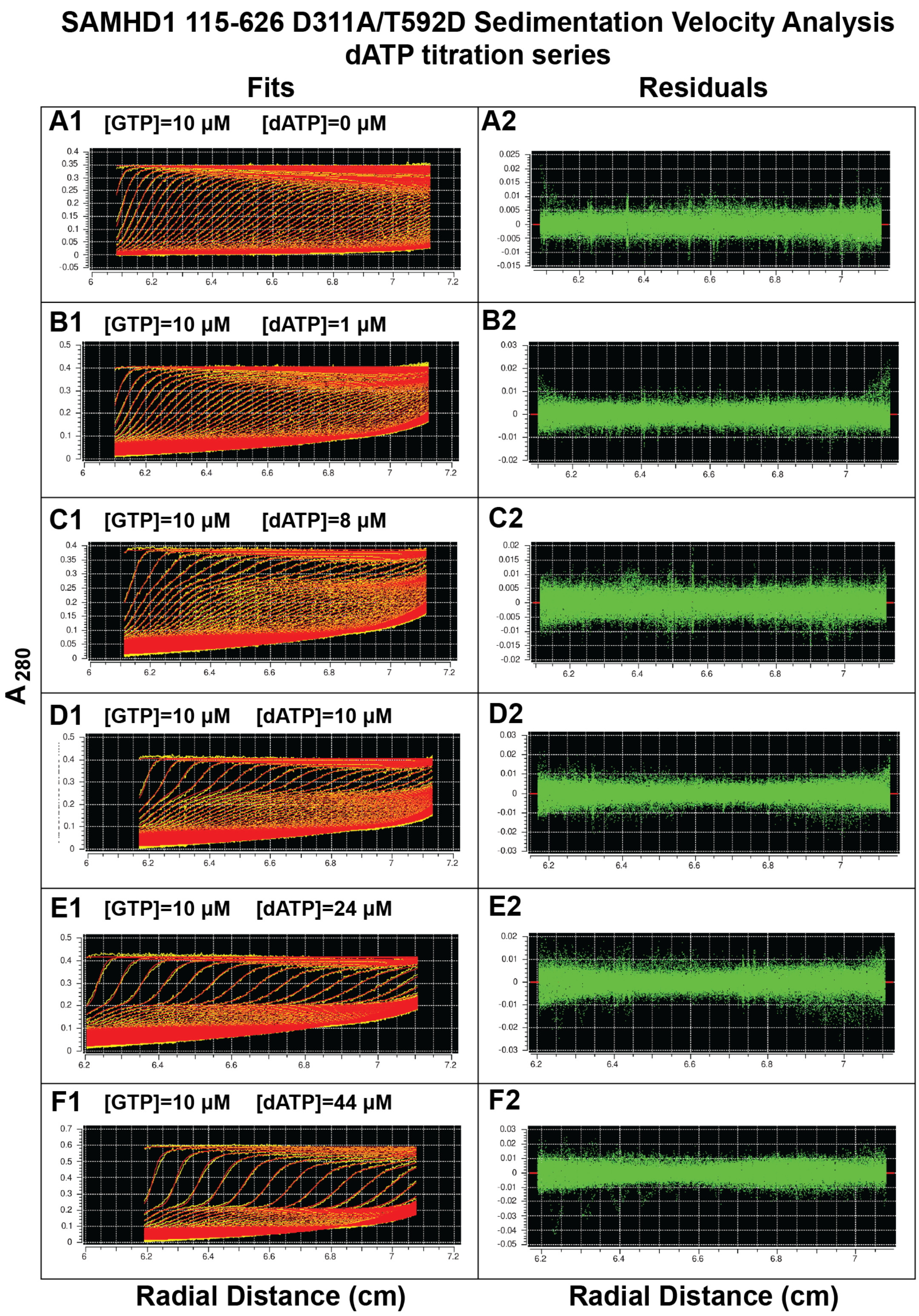

Figure S6. Sedimentation velocity analysis of SAMHD1 115-626 D311A/T592D. dATP titration series. Left column shows fits of the 2D spectrum analysis to the experimental data. Right column shows residuals for the fits shown in the left column.

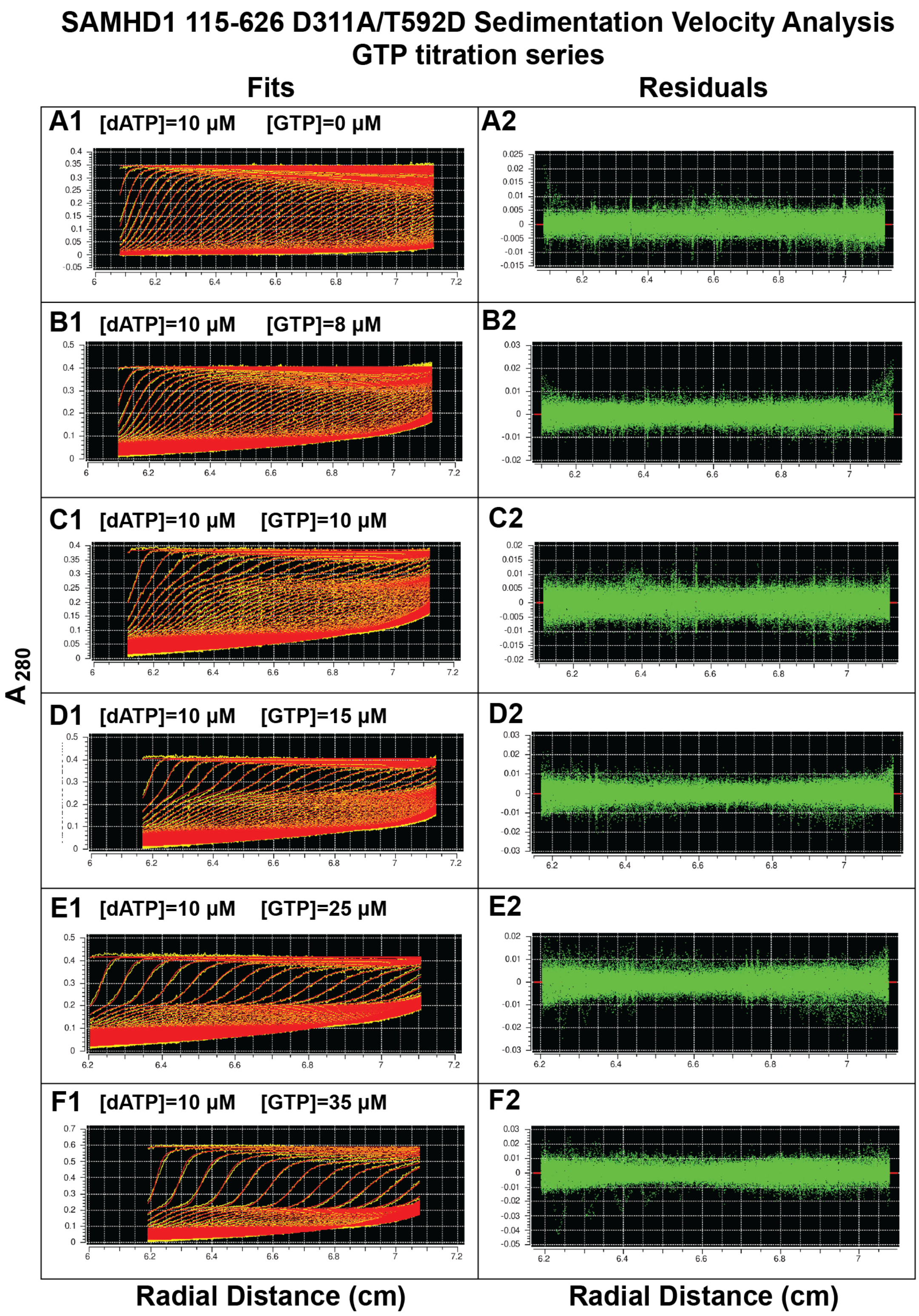

Figure S7. Sedimentation velocity analysis of SAMHD1 115-626 D311A/T592D. GTP titration series. Left column shows fits of the 2D spectrum analysis to the experimental data. Right column shows residuals for the fits shown in the left column.

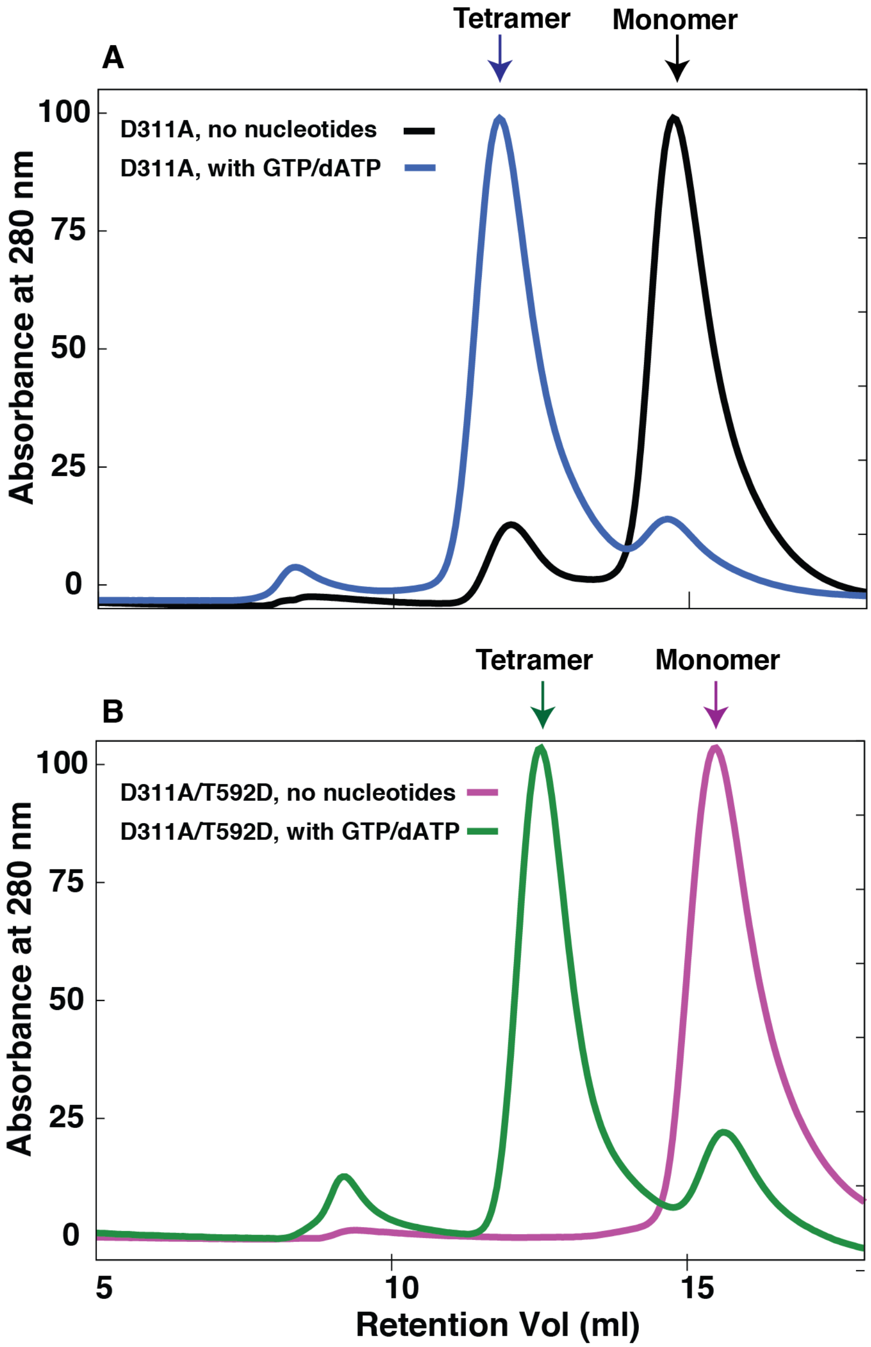

Figure S8. Size-exclusion chromatography analysis of SAMHD1 tetramerization. Size exclusion chromatography on the GE Superdex 10/300 GL column was used as an independent experimental method to verify results obtained by analytical ultracentrifugation. Tetramerization propensity of SAMHD1 115-626 D311A (**A**) in the presence of 25 μM GTP and 50 μM dATP in the running buffer is very similar to that of the SAMHD1 115-626 D311A/T592D double mutant (**B**). These results further support our findings that the T592D mutation does not have a significant effect on SAMHD1 tetramerization.

Supplementary Table S1: Statistics of the Analytical Ultracentrifugation Data Analysis for SAMHD1 115-626 constructs D311A and D311A/T592D

Protein	[GTP] µM	[dATP] µM	RMSD (A280 units)	Signal Amplitude (A280 units)	RMSD (in % units)	Figure # for fits	Figure # for residuals
D311A	10	0	0.00362332	0.3	1.2	S4.A1	S4.A2
D311A	10	10	0.00435893	0.45	1.0	S4.B1	S4.B2
D311A	10	15	0.00435288	0.475	0.9	S4.C1	S4.C2
D311A	10	20	0.00404862	0.48	0.8	S4.D1	S4.D2
D311A	10	30	0.00306953	0.49	0.6	S4.E1	S4.E2
D311A	10	80	0.00436204	0.5	0.9	S4.F1	S4.F2
D311A	0	10	0.00315321	0.3	1.1	S5.A1	S5.A2
D311A	6	10	0.00385161	0.31	1.2	S5.B1	S5.B2
D311A	10	10	0.00518438	0.4	1.3	S5.C1	S5.C2
D311A	12	10	0.00554935	0.34	1.6	S5.D1	S5.D2
D311A	20	10	0.00546419	0.45	1.2	S5.E1	S5.E2
D311A	50	10	0.0103151	0.62	1.7	S5.F1	S5.F2
D311A/T592D	10	0	0.00255047	0.35	0.7	S6.A1	S6.A2
D311A/T592D	10	1	0.00289906	0.37	0.8	S6.B1	S6.B2
D311A/T592D	10	8	0.00306009	0.38	0.8	S6.C1	S6.C2
D311A/T592D	10	10	0.00346852	0.4	0.9	S6.D1	S6.D2
D311A/T592D	10	24	0.00356995	0.42	0.8	S6.E1	S6.E2
D311A/T592D	10	44	0.00464987	0.6	0.8	S6.F1	S6.F2
D311A/T592D	0	10	0.00255047	0.35	0.7	S7.A1	S7.A2
D311A/T592D	8	10	0.00387097	0.44	0.9	S7.B1	S7.B2
D311A/T592D	10	10	0.00379624	0.33	1.2	S7.C1	S7.C2
D311A/T592D	15	10	0.00674489	0.55	1.2	S7.D1	S7.D2
D311A/T592D	25	10	0.00533047	0.6	0.9	S7.E1	S7.E2
D311A/T592D	35	10	0.00680763	0.65	1.0	S7.F1	S7.F2
